# Targeting the melanoma-associated antigen CSPG4 with HLA-C*07:01-restricted T-cell receptors

**DOI:** 10.3389/fimmu.2023.1245559

**Published:** 2023-10-02

**Authors:** Korbinian N. Kropp, Martina Fatho, Enes Huduti, Marilena Faust, Silke Lübcke, Volker Lennerz, Annette Paschen, Matthias Theobald, Thomas Wölfel, Catherine Wölfel

**Affiliations:** ^1^ Internal Medicine III, University Cancer Center (UCT), Research Center for Immunotherapy (FZI), University Medical Center (UMC) of the Johannes Gutenberg University and German Cancer Consortium (DKTK), Partner Site Frankfurt/Mainz, Mainz, Germany; ^2^ Dermatology, University Hospital, University Duisburg/Essen and German Cancer Research Consortium (DKTK), Partner Site Essen/Duesseldorf, Essen, Germany

**Keywords:** tumor immunotherapy, T cell receptor (TCR), CSPG4, chondroitin sulfate proteoglycan 4, TCR hemichain dominance

## Abstract

**Intorduction:**

Chondroitin sulfate proteoglycan 4 (CSPG4), also known as high molecular weight-melanoma associated antigen, is expressed in melanoma but also other tumor entities and constitutes an attractive target for immunotherapeutic approaches. While recent preclinical reports focused on anti-CSPG4 chimeric antigen receptors (CAR), we here explore T-cell receptor (TCR)-based approaches targeting CSPG4.

**Methods:**

The TCRs of two CSPG4-reactive T-cell clones (11C/73 and 2C/165) restricted by the highly prevalent HLA-C*07:01 allele were isolated and the respective αβTCR pairs were retrovirally expressed in CRISPR/Cas9-edited TCR-knockout T cells for functional testing. We also combined alpha and beta TCR chains derived from 11C/73 and 2C/165 in a cross-over fashion to assess for hemichain dominance. CSPG4^+^ melanoma, glioblastoma and lung cancer cell lines were identified and, if negative, retrovirally transduced with HLA-C*07:01.

**Results:**

Functional tests confirmed specific recognition of CSPG4^+^HLA-C*07:01^+^ target cells by the αβTCR retrieved from the parental T-cell clones and in part also by the cross-over TCR construct 2Cα-11Cβ. Despite high surface expression, the 11Cα-2Cβ combination, however, was not functional.

**Discussion:**

Collectively, 11C/73- and 2C/165-expressing T cells specifically and efficiently recognized CSPG4^+^HLA-C*07:01^+^ cancer cells which warrants further preclinical and clinical evaluation of these TCRs.

## 1 Introduction

Transfer of autologous *ex vivo* expanded tumor-infiltrating lymphocytes (TIL) comprising tumor-reactive CD4^+^ or CD8^+^ T cells has generated durable responses also in late-stage cancer patients (1, 2). Although isolation of TILs from surgically removed tumor tissue is highly efficient, *in vitro* expansion of functional tumor-reactive TILs is not always successful (3). This obstacle can be overcome by cloning of tumor-reactive T-cell receptors (TCRs) from sources such as TILs or patient-derived peripheral blood mononuclear cells (PBMCs) and subsequent TCR transfer into autologous *ex vivo* expanded T cells by retroviral transduction (4). Tumor-reactive TCRs can recognize a variety of different antigen classes such as neoantigens arising from patient-specific mutations or tumor-associated antigens (TAA) (5). TAAs are overexpressed in cancers but not or only minimally expressed in normal tissue, and, in contrast, to patient-specific neoantigens, are often shared among patients and tumor entities (6). The immunotherapeutic potential and safety of targeting TAAs such as NY-ESO-1, MAGE-A3, Melan-A/MART-1 or gp100 has been shown for multiple entities both in preclinical and clinical studies (4, 7–9). In our analysis of T-cell responses in the previously described patient model Ma-Mel-86 (10), we have identified two melanoma-reactive CD8^+^ T-cell clones, 11C/73 (11) and 2C/165, targeting the TAA chondroitin sulfate proteoglycan 4 (CSPG4). These T-cell clones were established by limiting dilution of tumor-reactive mixed lymphocyte-tumor cultures (MLTCs) set up with the patient-derived cell line Ma-Mel-86c and autologous PBMCs. Screening of patient-derived cDNA libraries uncovered CSPG4 as the specific target of both T-cell clones. Subsequent experiments revealed the HLA-C*07:01-restricted 9-mer HIIFPHGSL and 10-mer PHIIFPHGSL to be the exact target peptides. The HLA-C*07:01 allele is highly prevalent (12) and CSPG4 represents an optimal immunotherapeutic target due to its consistent upregulation in melanoma, and low/absent expression in healthy tissue (13). Of note CSPG4-reactive CD4^+^ T cells have been identified in melanoma patients before (14, 15). Moreover, transfer of anti-CSPG4 chimeric-antigen receptor (CAR)-T cells eradicated established melanomas in mice (16, 17). In addition, expression of CSPG4 is not restricted to melanoma, but was also detected in various other cancer entities (17), and transfer of anti-CSPG4 CAR T cells controlled tumor growth in models of breast cancer, head and neck cancer, glioblastoma and sarcoma (17–19).

Given these encouraging preclinical reports on CSPG4 as an immunotherapeutic target, we set out to further characterize the two previously identified HLA-C*07:01-restricted melanoma reactive T-cell clones 11C/73 and 2C/165 directed against the TAA CSPG4.

## 2 Materials and methods

### 2.1 Cell lines

K562 cells were provided by Alexander Knuth (affiliated with the University Medical Center Mainz, Mainz, Germany, at the time of material transfer), HEK293T cells were provided by Nilabh Shastri (UC Berkeley, Berkeley, CA, USA) and Phoenix-Ampho cells were provided by Matthias Theobald (University Medical Center Mainz, Mainz, Germany). Patient-derived cell lines were established with informed consent and in accordance with the local ethical guidelines. Melanoma lines were A375, provided by Krishnaraj Rajalingam (University Medical Center Mainz, Mainz, Germany), D03, D05, D10, D14, D17, D18, D22, D28, D41, provided by Chris W. Schmidt (Queensland Institute of Medical Research, Brisbane, Australia), Ma-Mel-66b, Ma-Mel-86b, Ma-Mel-86c, provided by Anette Paschen (Department of Dermatology, University Medical Center Essen, Essen, Germany), MZ7-Mel#1, provided by Thomas Wölfel (University Medical Center Mainz, Mainz, Germany). Glioblastoma cell lines MZ-219-GBM, MZ-221-GBM, MZ-222-GBM, MZ-257-GBM, MZ-304-GBM, MZ-373-GBM, MZ-415-GBM, MZ-416-GBM, MZ-483-GBM and lung cancer cell lines LC-MZ-1, LC-MZ-6, LC-MZ-16 were provided by Sigrid Horn (University Medical Center Mainz, Mainz, Germany). Lung cancer cell line NW-BC-11 was provided by Elke Jäger (Northwest Hospital, Frankfurt/Main, Germany). Renal cell cancer cell lines RCC 1170, RCC 1774, RCC 1795, RCC 1846, RCC 1851, RCC 1879, RCC 1940, RCC 1973, RCC FM-KOT, RCC PB5, RCC PB6, RCC PB7 were provided by Barbara Seliger (University Medical Center Halle, Halle/Saale, Germany). Phoenix-Ampho cells were maintained in DMEM (Thermo Fisher Scientific, Waltham, MA, USA) supplemented with 10% fetal calf serum (FCS; PAN Biotech, Aidenbach, Germany), 1% Penicillin/Streptomycin, 1% L-Glutamine (Sigma-Aldrich, St. Louis, MO, USA) and 25 mM HEPES buffer (Lonza, Basel, Switzerland). All other cell lines were maintained in RPMI (Thermo Fisher Scientific) supplemented with 10% FCS and 1% Penicillin/Streptomycin. T cells were maintained in Panserin 413 medium (PAN Biotech, Aidenbach, Germany) supplemented with 10% human serum (kindly provided by the blood bank of the University Medical Center Mainz, Mainz, Germany) and 1% Penicillin/Streptomycin. All cells were kept at 37°C in a humidified atmosphere.

### 2.2 Culture of parental T-cell clones

The T-cell clones 11C/73 and 2C/165 were weekly restimulated with irradiated (100 Gy) antigen-expressing tumor cells (Ma-Mel-86c) at a stimulator-to-T cell ratio of 1:10 in the presence of 250 IU/mL IL-2 (Novartis, Basel, Switzerland).

### 2.3 Isolation and stimulation of T cells

PBMCs were isolated by Ficoll gradient centrifugation from buffy coats of healthy donors obtained from the blood bank of the University Medical Center of the Johannes-Gutenberg university Mainz. CD4^+^ or CD8^+^ T cells were isolated from PBMCs by positive selection using magnetic bead separation (Milteny, Bergisch Gladbach, Germany) and stimulated for 48 h with the anti-CD3 mAb OKT3 (30 ng/mL) (Milteny) in the presence of 600 IU/ml IL-2 (Novartis) and the irradiated (100 Gy) cells of the flow-through of the isolation procedure.

### 2.4 Knockout of endogenous TCRs using CRISPR/Cas9

Stimulated CD4^+^ or CD8^+^ T cells were washed twice with PBS and once with Opti-MEM (Thermo Fisher Scientific) and were subsequently resuspended in 100 µL supplemented Nucleofector Solution (Lonza, Basel, Switzerland) per cuvette at a concentration of 4-5e6 T cells/100 µL. In parallel, RNPs targeting the *TRAC* (TCAGGGTTCTGGATATCTGT) and *TRBC1/2* [TGGCTCAAACACAGCGACCT (20)] locus were formed. First, 0.6 µL crRNA (200 µM; IDT, Coralville, IA, USA) and 0.6 µL tracrRNA (200 µM; IDT) were duplexed per electroporation cuvette for 5 min at 95°C. Thereafter, 1.7 µL of Cas9 protein (61 µM; IDT) and 2.1 µL Opti-MEM was added and RNPs were generated by incubation for 20 minutes at room temperature. Thereafter, the T-cell suspension was mixed with the RNPs and 1 µL electroporation enhancer (100 µM; IDT), transferred to an 100 µL electroporation cuvette and immediately electroporated using program T-023 on a Nucleofector 2B device (Lonza). Next, 500 µL prewarmed T-cell medium was added to electroporated T cells and cells were rested for 5 min at 37°C. Subsequently, T cells were transferred to 24-well plates and cultured for 7 days in the presence of IL-2 (600 IU/mL) prior to retroviral transduction.

### 2.5 T-cell transduction

In brief, Phoenix amphotropic retroviral packaging cells were seeded at a density of 1.3 e6 cells per 100 mm plate. The next day, packaging cells were cotransfected with 5 µg of each of the helper plasmids pCOLT-GALV and pHIT60, and 10 µg of the retroviral pMX vector encoding for the TCR expression constructs using Fugene 6 (Promega, Madison, WI, USA) according to the manufacturer’s instructions. On the following day medium was changed to T cell medium and virus soup was harvested after additional incubation for 16 h by pelleting cellular debris. Subsequently, 1-2 e6 stimulated CRISPR/Cas9-edited T cells were spin infected with 1 mL of virus soup (90 min at 2000 rpm) in the presence of Polybrene (5 μg/ml; Sigma Aldrich) and IL-2 (600 U/mL; Novartis) and were further cultivated for 22 h. On the day after transduction infected T cells were restimulated with anti-CD3/CD28 beads (Milteny Biotec) and were selected with puromycin (1 μg/mL; Sigma Aldrich). After 7 days, transgenic T cells were incubated for 5 min on a magnet to remove anti-CD3/CD28 beads.

### 2.6 Plasmids and cloning

TCR expression constructs were generated as previously described (21). HLA-C*07:01, HLA-C*07:02 cDNA, as well as truncated *CSPG4* cDNA ending with codon 562 were cloned into pcDNA3.1/V5-His TOPO (Invitrogen, Waltham, MA, USA) using standard cloning techniques as previously described (22). Plasmids were propagated in 10-beta competent E. coli (NEB, Ipswich, MA, USA) and were purified using the Midi or Maxi Plasmid Prep kits from Qiagen (Hilden, Germany).

### 2.7 Flow cytometry

For surface staining of tumor or T cells, cells were washed once with PBS and subsequently stained for 30 minutes at 4°C in the dark with the indicated surface antibodies diluted in PBS supplemented with 0.1% bovine serum albumin and 2.5 mM EDTA (FACS buffer). The following monoclonal antibodies were used: CD4-PE (clone 13B8.2; Beckman Coulter, Brea, CA, USA), CD8-APC (clone B9.11; Beckman Coulter), CD8-PE (clone B9.11; Beckman Coulter), anti-murine TCR-FITC (clone H57-697; Biolegend, San Diego, CA, USA), anti-human TCR-APC (clone IP26, Biolegend) and CSPG4-PE (REA1041, Milteny). For surface staining of MHC-I, tumor cells were stained by incubation with supernatant of the hybridoma W6/32 (kindly provided by Dr. P. Parham, Departments of Structural Biology and Microbiology and Immunology, Stanford University, Stanford, CA, USA) followed by staining with polyclonal goat-anti-mouse-FITC (Biozol, Eching, Germany). Flow cytometry was performed on a FACS Canto II (BD Bioscience, San José, CA, USA) and data was analyzed using FlowJo (BD Bioscience, Version 10.7).

### 2.8 Bioluminescence assay

Cytotoxicity of T-cell clones and transgenic T cells was analyzed by a bioluminescence-based lysis assay as recently described (21). In brief, Ma-Mel-86b and Ma-Mel-86c were engineered to express Firefly luciferase (Fluc) and cocultured with effector cells at an E:T ratio of 10:1 in the presence of 0.15 mg/mL D-Luciferin (Biosynth, St. Gallen, Switzerland). Subsequently, relative luminescence units (RLU) were determined over time in 3 h intervals using a FluoStar Omega plate reader (BMG Labtech, Ortenberg, Germany) and a 10 s integration time. Spontaneous cell death was measured in wells containing target cells only, maximum cell death was induced by exposure of target cells to Digitonin (Sigma) at a concentration of 30 µg/mL. Lysis was calculated using the following equation: lysis [%] = 100*[(spontaneous RLU—test unit RLU)/(spontaneous RLU—maximum RLU)].

### 2.9 IFNγ-ELISpot assays

IFNγ-ELISpot assays were performed as previously described (21). In brief, 293T (20,000 cells/well) were transfected with cDNA encoding HLA-C*07:01 or HLA-C*07:02 and either cotransfected with cDNA encoding truncated *CSPG4* ending with codon 562 or pulsed with the CSPG4-derived peptides HIIFPHGSL (9-mer) or PHIIFPHGSL (10-mer) (both synthesized by Dr. Jan-Wouter Drijfhout, University Medical Center Leiden, The Netherlands) 24h after transfection of HLA-encoding cDNAs. Transfection was performed directly on ELISpot plates (Millipore, Burlington, MA, USA) using Lipofectamine 2000 (Invitrogen, Carlsbad, CA, USA) according to the manufacturer´s recommendations. Parental T-cell clones or transgenic T cells (1000 – 10,000 (c)TCR^+^ cells/well) were then added to (peptide-pulsed) transfectants. In some experiments T cells were directly added to freshly seeded tumor cells (40,000 – 50,000 cells/well). After 20–24 h ELISpot plates were developed and IFNγ spots were visualized with an ImmunoSpot analyzer (Cellular Technology Limited, Cleveland, OH, USA).

### 2.10 Statistics

Students t-test or one-way ANOVA was used to calculate statistical significance and tests were considered significant when the p value was < 0.05. The peptide concentration necessary to elicit a half-maximal response in ELISpot assays (pEC50) was calculated by performing non-linear regression using a variable slope model with four parameters. Data was analyzed in GraphPad Prism (Version 9.0.0). All experiments were independently performed at least twice.

## 3 Results

### 3.1 Characterization of two distinct anti-CSPG4 CD8^+^ T-cell clones restricted by HLA-C*07:01 and isolation of their αβTCR sequences

**Figure 1:**
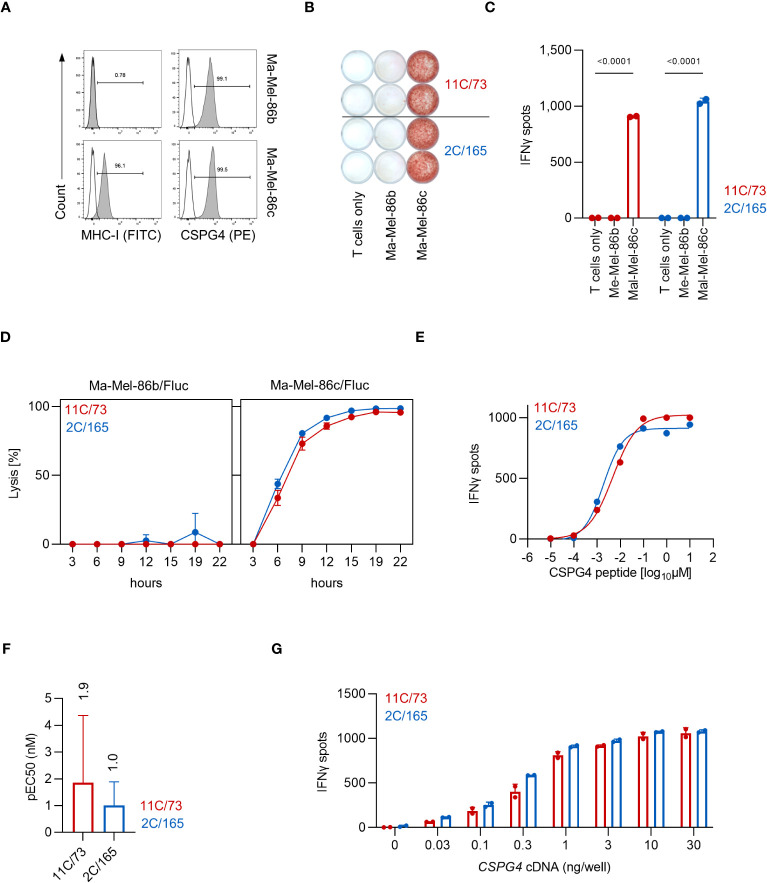
The T-cell clones 11C/73 and 2C/165 recognize CSPG4 and are restricted by HLA-C*07:01. **(A)** Surface expression of CSPG4 and MHC-I on the indicated cell line was measured by flow cytometry. Line: unstained, filled: stained with an anti-CSPG4 or anti-MHC-I mAb. **(B, C)** T-cell clones (5.000 cells/well) were co-cultured with Ma-Mel-86c (HLA-C*07:01^+^CSPG4^+^) or Ma-Mel-86b (MHC-I^-^CSPG4^+^) (50.000 cells/well). IFNγ production was measured by ELISpot. Quantification of **(B)** is shown in **(C)**. **(D)** Lysis of Fluc tumor targets by the T-cell clones was assessed over time at an E:T ratio of 10:1 in a bioluminescence-based cytolysis assay. Data points are mean of three technical replicates. Error bars indicate SD. **(E–G)** T-cell clones (5.000 cells/well) were co-cultured for 24 h with 293T cells (20.000 cells/well) transiently transfected with HLA-C*07:01 cDNA and pulsed with **(E, F)** CSPG4:554-562 peptide or **(G)** co-transfected with *CSPG4:1-562* cDNA. IFNγ production was measured by ELISpot. Data points are mean of two technical replicates. Error bars indicate SD. **(F)** pEC50 determined by peptide titration. Data points are mean pEC50 values of three independent experiments. Error bars indicate SD.

We started our initial analyses of the HLA-C*07:01-restricted T-cell clones 11C/73 and 2C/165 using the cell lines Ma-Mel-86b and Ma-Mel-86c which had been established from distinct metastases of patient Ma-Mel-86 (10). HLA-C*07:01 is expressed in both cell lines, but in contrast to Ma-Mel-86c cells, Ma-Mel-86b cells lack surface expression of MHC-I due to biallelic loss of *B2M* [(10) and **Figure 1A**]. Both cell lines are CSPG4^+^ as evidenced by surface staining (**Figure 1A**). Co-culture of the T-cell clones 11C/73 and 2C/165 with the HLA-C*07:01^+^CSPG4^+^ cell line Ma-Mel-86c resulted in release of IFNγ, while no relevant IFNγ-production was observed upon co-culture with the MHC-I^-^CSPG4^+^ cell line Ma-Mel-86b in IFNγ-ELISpot assays (**Figures 1B, C**). The killing capacity of the T-cell clones was assessed in a bioluminescence-based cytotoxicity assay. Both T-cell clones efficiently lysed the HLA-C*07:01^+^CSPG4^+^ cell line Ma-Mel-86c/Fluc but not the control cell line Ma-Mel-86b/Fluc (**Figure 1D**). To compare functional avidity, we employed the T-cell clones against HLA-C*07:01 cDNA-transfected 293T cells loaded with titrated amounts of the CSPG4:554-562 peptide (9-mer). As shown in **Figure 1E**, both T-cell clones recognized peptide-loaded target cells in a concentration-dependent manner, displayed comparable and high functional avidities, and similar maximum IFNγ release. We found the concentration of peptide necessary for a half maximal response (pEC50) for T-cell clone 2C/165 to be slightly but not significantly lower compared to T-cell clone 11C/73 (**Figure 1F**). While we focused our analysis on the 9-mer CSPG4:554-562 peptide, we found that the 10-mer CSPG4 peptide CSPG4:553-562 was also recognized by both T-cell clones albeit with a lower functional avidity as compared to the 9-mer (**Supplementary Figure 1**). For further validation and as a reference point for later experiments, we also extended our analyses to recognition of endogenously processed antigen and co-transfected 293T cells with both HLA-C*07:01 cDNA and titrated amounts of a *CSPG4* cDNA fragment containing the peptide-coding region (codon 1-562). Both T-cell clones recognized *CSPG4:1-562* cDNA-transfected targets comparably and in a dose dependent manner (**Figure 1G**).

**Figure 2:**
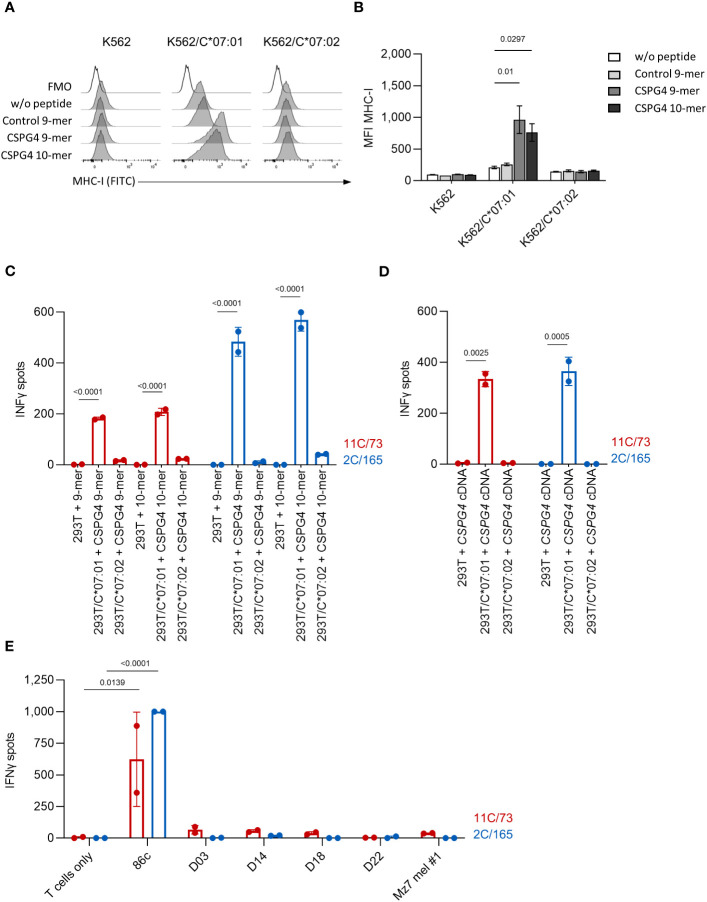
The T-cell clones 11C/73 and 2C/165 show no reactivity against HLA-C*07:02^+^ targets. **(A, B)** K562 (HLA-C*03:04,05:01) or K562 stably expressing either HLA-C*07:01 or HLA-C*07:02 were incubated with the indicated peptide at 100 µg/mL and subsequently analyzed for MHC-I expression by flow cytometry. **(A)** Exemplary histogram for each condition. **(B)** Quantification of median fluorescence intensity (MFI). **(C, D)** The indicated T-cell clones (5.000 cells/well) were co-cultured with 293T cells (20,000 cells/well) transiently transfected with either HLA-C*07:01 or HLA-C*07:02 cDNA and pulsed either with **(C)** CSPG4-derived peptides or **(D)** cotransfected with *CPSG4:1-562* cDNA. **(E)** The indicated T-cell clones (20.000 cells/well) were co-cultured with the indicated HLA-C*07:01^+^ (Ma-Mel-86c) or HLA-C*07:02^+^ (D03, D14, D18, D22, Mz7 mel #1) tumor cell lines (40.000 cells/well). IFNγ production was measured by ELISpot. Data points are mean of two technical replicates. Error bars indicate SD.

Among HLA-C alleles, HLA-C*07:02 displays a high homology to HLA-C*07:01 (99.4%) and only differs in two residues (90 and 123) (23). To test whether the immunogenic CSPG4 peptides can also bind to HLA-C*07:02, we pulsed wildtype K562 cells (HLA-C*03:04, 05:01) or K562 cells stably expressing HLA-C*07:01 or HLA-C*07:02 with CSPG4:553-562 (9-mer), CSPG4:554-562 (10-mer) or a control peptide presented by HLA-B*15:01 [HERPUD1^G161S^:154-162 (22)] and analyzed MHC-I levels by flow cytometry. Both CSPG4:553-562 and CSPG4:554-562 increased the expression levels of MHC-I in K562/HLA-C*07:01 but not in K562 wildtype or K562/C*07:02 cells. (**Figures 2A, B**). Next, we co-cultured the T-cell clones with either 293T cells transfected with HLA-C*07:01 or HLA-C*07:02 cDNA and loaded with the CSPG4:554-562 (9-mer) or CSPG4:553-562 (10-mer) peptide (**Figure 2C**) or cotransfected with *CSPG4:1-562* cDNA (**Figure 2D**) or co-cultured them with HLA-C*07:02^+^CSPG4^+^ melanoma cell lines (**Figure 2E**). In contrast to HLA-C*07:01-expressing target cells, we did not observe reactivity of the T-cell clones against HLA-C*07:02^+^ target cells suggesting no relevant overlap in CSPG4 peptide binding among these highly homologous HLA-C alleles.

Given the high functional avidity and confirmed specificity of both T-cell clones, we next employed the 5’-RACE method (24) to identify their TCR sequences. Using this approach, two αβTCRs were amplified from cDNA and Sanger Sequencing revealed distinct clonotypes. The combination of V(D)J alleles and CDR3 sequences are summarized in **Table 1**.

**Table 1:** Table summarizing the V(D)J usage and unique CDR3 sequences of anti-CSPG4 T-cell clones.

T-cell clone	TCR chain	V(D)J usage	CDR3 sequence
11C/73	α-chain	TRAV12-2*02/TRAJ45*01	CAVNAAGGGADGLTF
	β-chain	TRBV28*01/TRBD1*01/TRBJ2-2*01	CASSYDWGGELFF
2C/165	α-chain	TRAV35*02/TRAJ34*01	CAGSYNTDKLIF
	β-chain	TRBV3-1*01/TRBD2*01/TRBJ2-1*01	CASSQGWTRNEQFF

### 3.2 The 11Cαβ and 2Cαβ TCRs, and the cross-over TCR 2Cα-11Cβ recognize HLA-C*07:01/CSPG4

**Figure 3:**
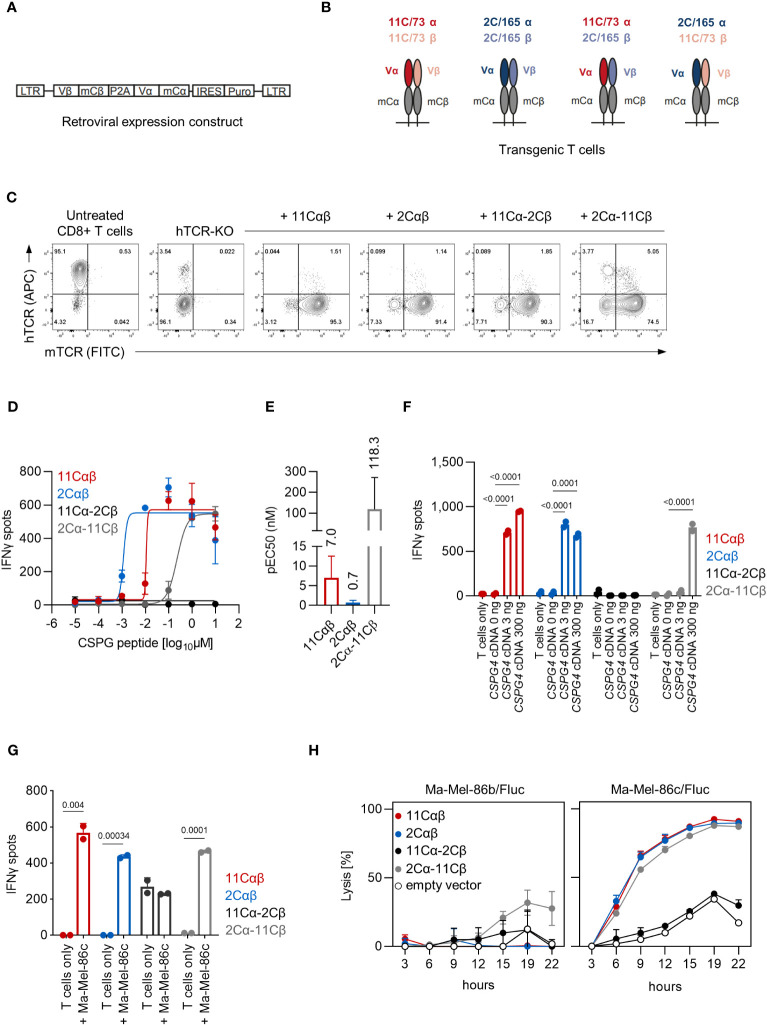
Functional analysis of TCR-transduced CD8^+^ T cells. **(A)** Schematic depiction of the retroviral cTCR expression construct. **(B)** Schematic depiction of the investigated combinations of the variable chains derived of the 11C/73 and 2C/165 T-cell clones. **(C)** Expression of cTCR and loss of hTCR expression in *TRAC-* and *TRBC1/2*-edited transgenic CD8^+^ T cells as determined by flow cytometry. **(D–G)** The indicated transgenic CD8^+^ T-cell populations (10,000 TCR^+^ cells/well) were co-cultured with **(D–F)** 293T cells (20,000 cells/well) transiently transfected with HLA-C*07:01 cDNA and pulsed with **(D, E)** CSPG4:554-562 peptide or **(F)** co-transfected with *CSPG4:1-562* cDNA, or **(G)** were co-cocultured with Ma-Mel-86c (HLA-C*07:01^+^CSPG4^+^) cells (50,000 cells/well). IFNγ production was measured by ELISpot. Data points are mean of two technical replicates. Error bars indicate SD. **(E)** pEC50 determined by peptide titration. Data points are mean pEC50 values of two independent experiments. Error bars indicate SD. **(H)** Lysis of Fluc tumor targets by transgenic CD8^+^ T cells was assessed over time at an E:T ratio of 10:1 in a bioluminescence-based cytolysis assay. Data points are mean of three technical replicates. Error bars indicate SD.

The αβTCR sequences of both clonotypes were subsequently cloned into retroviral expression constructs shown in **Figure 3A** to test whether PBMC-derived TCR-transduced CD8^+^ T cells can be redirected against CSPG4. Notably, our TCR-expression-constructs incorporated optimizations aimed to reduced mispairing of transgenic TCRs with the endogenous TCRs such as replacing the human constant regions with their murine counterparts (chimerized TCR; cTCR). To further reduce any residual TCR mispairing, expression of the endogenous TCRs was abrogated by electroporation of CRISPR/Cas9 ribonucleoproteins (RNPs) targeting the *TRAC* and *TRBC1/2* loci. Following human TCR (hTCR) knockout, TCR-KO CD8^+^ T cells were retrovirally transduced with the cTCR expression constructs and were expanded by weekly stimulation with anti-CD3/CD28 beads in the presence of IL-2. Of note, in addition to transducing the parental αβTCR gene-pairs, we also combined the alpha and beta chains of the 11C/73- and 2C/165-TCRs in a cross-over fashion to assess for potential TCR hemichain dominance (**Figure 3B**). As shown in **Figure 3C**, transgenic T cells highly expressed the transgenic cTCRs including the cross-over cTCRs as evidenced by staining for the murine constant region while retaining a TCR-knockout phenotype. After validating cTCR surface expression, transgenic T cells were tested in IFNγ-ELISpot assays against HLA-C*07:01 cDNA-transfected 293T cells pulsed with titrated amounts of CSPG4:554-562 peptide. Transgenic T cells expressing the 11Cαβ and 2Cαβ cTCR constructs derived from the respective parental 11C/73 and 2C/165 T-cell clone displayed similar maximal IFNγ-spot counts at high concentration of the CSPG4:554-562 peptide, but the 2Cαβ cTCR showed a higher functional avidity (**Figure 3D**). Interestingly, CD8^+^ T cells transduced with the cross-over construct 2Cα-11Cβ also recognized CSPG4:554-562 peptide-pulsed targets albeit with lower functional avidity despite similar maximal IFNγ-spot counts at high peptide concentration. These observations were also reflected in different pEC50 values which, however, did not reach statistical significance (**Figure 3E**). In contrast, no reactivity was observed with 11Cα-2Cβ cTCR-transduced CD8^+^ T cells even at high peptide concentrations. None of the employed T-cell populations released relevant amounts of IFNγ alone or in the presence of unpulsed HLA-C*07:01 + 293T cells confirming specificity of the T-cell responses (not shown).

Next, we extended our analyses to recognition of endogenously processed antigen and co-transfected 293T cells with both HLA-C*07:01 and two different amounts of *CSPG4:1-562* cDNA. CD8^+^ T cells transduced with the 11Cαβ and 2Cαβ TCR constructs specifically recognized *CSPG4:1-562* cDNA-transfected targets (**Figure 3F**). Mirroring the peptide titrations experiments, we also observed reactivity of the cross-over 2Cα-11Cβ cTCR albeit only at 300 ng of *CSPG4:1-562* cDNA per well. Again, spot counts of the 11Cα-2Cβ cTCR-transduced CD8^+^ T cells were not above background level. Analogous results were also seen when we tested the T-cell populations against HLA-C*07:01^+^CSPG4^+^ Ma-Mel-86c cells (**Figure 3G**). Finally, we assessed killing capacity of the TCR-transgenic T cells in a bioluminescence-based cytotoxicity assay. CD8^+^ T cells transduced with either parental or the 2Cα-11Cβ cTCR efficiently lysed the HLA-C*07:01^+^CSPG4^+^ cell line Ma-Mel-86c/Fluc but not the control cell line Ma-Mel-86b/Fluc (**Figure 3H**). Lysis rates of 11Cα-2Cβ cTCR-transduced CD8^+^ T cells were not above control T cells transduced with empty vector. Thus, these results validated both functionality and specificity of our TCR expression constructs and suggested that either the 2Cαβ has a dominant TCR alpha or that the 11Cαβ TCR has a dominant TCR beta chain regarding epitope recognition.

### 3.3 The 11Cαβ and 2Cαβ TCRs are co-receptor independent, but the cross-over cTCR 2Cα-11Cβ is co-receptor dependent

**Figure 4:**
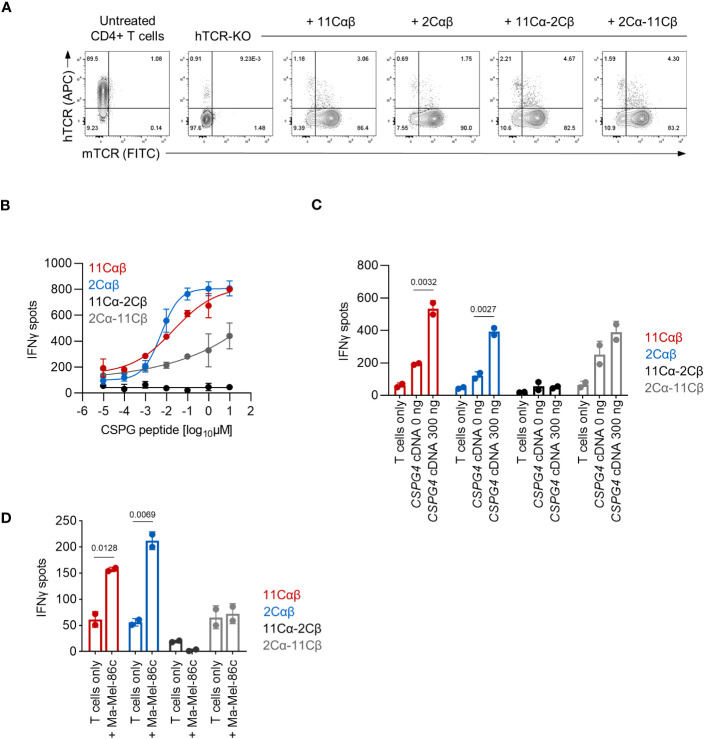
Functional analysis of TCR-transduced CD4^+^ T cells. **(A)** Expression of cTCR and loss of hTCR expression in *TRAC-* and *TRBC1/2*-edited transgenic CD4^+^ T cells as determined by flow cytometry. **(B–D)** The indicated transgenic CD4^+^ T-cell populations (10,000 TCR^+^ cells/well) were co-cultured with **(B, C)** 293T cells (20,000 cells/well) transiently transfected with HLA-C*07:01 cDNA and pulsed with **(B)** CSPG4:554-562 peptide or **(C)** co-transfected with *CSPG4:1-562* cDNA, or **(D)** were co-cocultured with Ma-Mel-86c (HLA-C*07:01^+^CSPG4^+^) cells (50,000 cells/well). IFNγ production was measured by ELISpot. Data points are mean of two technical replicates. Error bars indicate SD.

To analyze CD8 co-receptor dependence of the cTCR constructs for T cell activation, we generated PBMC-derived transgenic CD4^+^ T cells. The endogenous TCRs were again deleted by electroporation of CRISPR/Cas9 RNPs targeting both the *TRAC* and *TRBC1/2* loci (**Figure 4A**). We observed functionality of the 11Cαβ-cTCR, the 2Cαβ-cTCR and the cross-over construct 2Cα-11Cβ cTCR when expressed in CD4^+^ T cells and tested against HLA-C*07:01 cDNA-transfected 293T cells loaded with titrated amounts of CSPG4:554-562 peptide (**Figure 4B**). In contrast to our observations with transgenic CD8^+^ T cells, even at the highest peptide concentration tested, maximum IFNγ release of the cross-over 2Cα-11Cβ-cTCR construct was lower compared to the 11Cαβ- and 2Cαβ-cTCRs. The cross-over construct 11Cα-2Cβ was, again, non-functional. These results were also mirrored when transgenic CD4^+^ T cells were tested against 293T cells co-transfected with both HLA-C*07:01 and *CSPG4:1-562* cDNA (**Figure 4C**). However, in contrast to our observations with transgenic CD8^+^ T cells, CSPG4^+^ HLA-C*07:01^+^ Ma-Mel-86c cells were only recognized by the parental cTCRs (**Figure 4D**). Collectively these results suggested that T-cell activation mediated by the parental cTCRs was co-receptor independent. In contrast, the functional cross-over construct showed co-receptor dependence when tested against Ma-Mel-86c cells presumably reflecting its lower functional avidity.

### 3.4 Reactivity of anti-CSPG4 T cells against melanoma, glioblastoma and lung cancer cell lines

**Figure 5:**
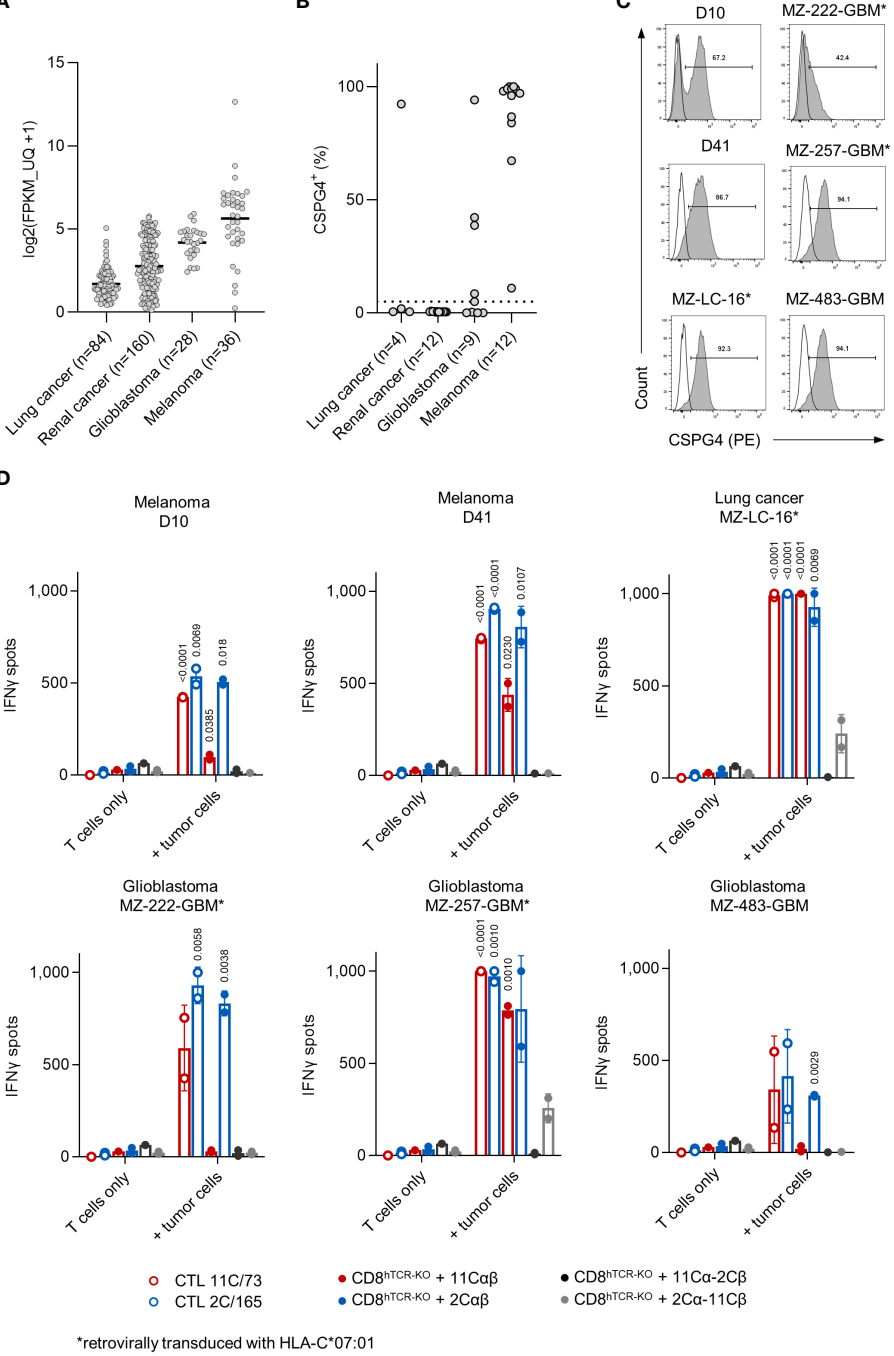
TCR-transduced CD8^+^ T cells recognize allogeneic CSPG4^+^ melanoma, glioblastoma and lung cancer cell lines. **(A)** *CSPG4* mRNA expression in the indicated entities obtained from the publicly available pan-cancer TCGA dataset (25) accessed through cBioPortal (26). **(B)** CSPG4 surface expression on melanoma (n=12) and glioblastoma (n=9), lung (n=4) and renal cancer (n=12) cell lines was determined by flow cytometry. **(C)** Histograms of CSPG4 expression in the indicated cell lines as determined by flow cytometry. Line: unstained, filled: stained with an anti-CSPG4 mAb. **(D)** Parental T-cell clones (10,000 cells/well) or hTCR-knockout CD8^+^ T cells transduced with the indicated cTCR construct (10,000 cTCR^+^ cells/well) were co-cultured with tumor targets (50,000 cells/well). IFNγ production was measured by ELISpot. Data points are mean of technical replicates. Error bars indicate SD.

According to the TCGA dataset high *CSGP4* mRNA expression is seen in melanoma, glioblastoma, renal and lung cancer (**Figure 5A**). We therefore tested CSPG4 surface expression in a panel of cancer cell lines derived from these entities (n=37). CSPG4 surface expression (above an arbitrary 5% threshold) was observed in 12/12 (100%) melanoma, 5/9 (56%) glioblastoma, 1/4 (25%) lung and 0/12 (0%) renal cancer cell lines (**Figure 5B**). Based on these results we selected a panel of CSPG4^+^ cell lines shown in **Figure 5C**. CSPG4^+^ but HLA-C*07:01^-^ cells lines were retrovirally transduced with HLA-C*07:01 (MZ-222-GBM, MZ-257-GBM and MZ-LC-16). We then tested the parental T-cell clones or CD8^+^ T cells expressing the indicated transgenic cTCR for recognition of our target panel in IFNγ-ELISpot assays. We observed high IFNγ spot counts with both T-cell clones against all cell lines tested with T-cell clone 2C/165 showing higher spot counts against D10, D41 and MZ-GBM-222 compared to T-cell clone 11C/73. Transgenic CD8^+^ T cells expressing the 11Cαβ- or 2Cαβ-cTCR elicited IFNγ spot formation above background levels when co-cultured with the endogenously HLA-C*07:01^+^ melanoma cell lines D10, D41, the glioblastoma cell line MZ-257-GBM/HLA-C*07:01 and the lung cancer cell line MZ-LC-16/HLA-C*07:01 (**Figure 5D**). 2Cαβ-cTCR-transduced T cells additionally recognized the glioblastoma cell line MZ-222-GBM/HLA-C*07:01 and the endogenously HLA-C*07:01^+^ glioblastoma cell line MZ-483-GBM. Of note, we also observed reactivity of the cross-over 2Cα-11Cβ-cTCR against the transfected cell lines MZ-257-GBM/HLA-C*07:01 and MZ-LC-16/HLA-C*07:01, while no reactivity was observed with the 11Cα-2Cβ-cTCR against any of the tested cell lines. Overall, IFNγ spot counts elicited by the 2Cαβ-cTCR were comparable to the IFNγ spot counts of both parental T-cell clones.

## 4 Discussion

Here, we report the identification and preclinical characterization of two distinct αβTCRs specific for the melanoma-associated antigen CSPG4 restricted by the highly prevalent HLA-C*07:01 allele (~30%). When expressed as chimerized TCR constructs in T cells both TCRs specifically recognized the CSPG4:554-562 peptide as well as endogenously processed CSPG4 with similar high functional avidity. CSPG4 has been previously investigated as an immunotherapeutic target for CAR T cells, among others in melanoma and glioblastoma (16–19), and we also observed specific recognition in a panel of CSPG4^+^/HLA-C*07:01^+^ melanoma and glioblastoma cell lines.

Previous preclinical studies indicated that knockout of the endogenous TCR using genome editing platforms such as TALEN (27) or CRISPR/Cas9 (20) substantially enhances TCR-expression and functionality of TCR-redirected T cells. Moreover, a recent first-in-human trial confirmed that adoptive transfer of T cells retrovirally transduced with a tumor-reactive TCR and edited at the *TRAC*, *TRBC* and *PDCD1* loci by CRISPR/Cas9 was safe and feasible (28). Similarly, we disrupted expression of the endogenous TCR prior to retroviral transduction of our TCR constructs by electroporation of CRISPR/Cas9 RNPs targeting *TRAC* and *TRBC1/2*. This procedure was highly efficient, and we observed only minimal residual expression of the endogenous TCRs. This is in line with previous reports (20) and validates that knockout of the endogenous TCR can easily be integrated into existing T-cell engineering pipelines. However, the TCR expression constructs herein were chimerized and contained murine instead of human constant-regions. This has been described to reduce mispairing with endogenous TCRs thereby increasing surface expression and enhancing functionality of transgenic TCRs (29, 30) without reports of adverse immune responses related to the murine sequences (31, 32). Beyond enhancing TCR surface expression, chimerization may also affect TCR/CD3 stability thereby independently increasing TCR functionality (29, 30). Whether chimerization of TCR expression constructs also enhances functionality in the absence of endogenous TCR chains remains to be investigated.

Our data on functional avidity in transgenic CD8^+^ T cells measured by pEC50 largely mirrored the results obtained with the parental T-cell clones although we observed a more pronounced difference in functional avidity of the 2Cαβ-cTCR as compared to the 11Cαβ-cTCR when tested in CD8^+^ T cells in contrast to the parental T-cell clones. By performing co-transfection of cDNA coding for HLA-C*07:01 and a *CSPG4* fragment containing the peptide-coding region, we confirmed that transgenic T cells were also able to recognize endogenously processed antigen. Recognition of the HLA-C*07:01^+^/CSPG4^+^ cell line Ma-Mel-86c further evidenced that transgenic T cells were also able to recognize the endogenously expressed and presented peptide. Beyond its well described uniform and high expression in melanoma, examination of the TCGA mRNA dataset showed high expression of *CSPG4* in a variety of tumor entities including melanoma, glioblastoma, lung and renal cancer (17). Screening of a panel of cell lines from these histologies found CSPG4 to be uniformly expressed in all tested melanoma cell lines as well as in a subset of glioblastoma and one lung cancer cell line. Strikingly, despite high *CSPG4* mRNA expression in the TCGA dataset none of the 12 tested renal cell cancer cell lines expressed CSPG4 as determined by flow cytometry. Testing of our transgenic CD8^+^ T cells confirmed recognition of these HLA-C*07:01^+^/CSPG4^+^ cell lines with the 2Cαβ-cTCR displaying superior functionality. Interestingly, while others reported identical functionality of T-cell clones and transgenic T cells expressing the parental TCRs (33), we found that our observations with the parental T-cell clones not fully translated to cTCR-transgenic T cells. However, a comparison of cTCR-transgenic T cells with the T-cell clones should be interpreted with caution due to the different developmental history of the parental blood-derived T-cell clones affecting all parameters beyond TCR affinity adding up to overall avidity.

We also extended our analyses to the CD4^+^ T-cell subset and found that transgenic CD4^+^ T cells were also able to recognize peptide-loaded target cells albeit with a lower functional avidity compared to transgenic CD8^+^ T cells. The co-receptor CD8 stabilizes TCR-pMHC (peptide-loaded MHC) interactions thereby increasing functional T-cell avidity (34). High-affinity MHC-I-restricted TCRs can, however, display CD8 co-receptor independence and mediate anti-tumor reactivity when expressed in CD4^+^ T cells (35, 36). This may not only be beneficial due to sustaining proliferation of CD8^+^ T cells by release of cytokines but can also involve direct cytotoxicity mediated by CD4^+^ T cells (36–38). In the present study, we observed IFNγ release upon co-culture of transgenic CD4^+^ T cells with target cells that were loaded with CSPG4 peptide, transfected with high concentrations of *CSPG4* cDNA and against CSPG4^+^HLA-C*07:01^+^ Ma-Mel-86c cells. From these experiments we concluded that both parental TCRs are co-receptor independent pointing towards a high affinity.

Interestingly, we observed that the cross-over cTCR construct 2Cα-11Cβ combining TCR alpha and beta chains from two distinct TCRs was functional when expressed in CD8^+^ T cells and in CD4^+^ T cell at high peptide concentrations. TCR alpha and beta chains usually cooperate to confer antigen-specificity and MHC restriction, but several studies have reported chain-centric TCRs, in which antigen-specificity is dominated by one of the TCR hemichains (39–41). Pairing of the dominant chain of these chain-centric TCRs with different counterchains was reported to generate functional TCRs with enhanced, reduced or abrogated TCR function without affecting their antigen specificity (40). In the present study, we found that the 2Cα-11Cβ TCR was functional and therefore hypothesize that either the 11C/73 TCR is a beta-chain centric or that the 2C/165 TCR is an alpha-chain centric TCR. Given that the 2Cα-11Cβ TCR displayed lower avidity in comparison to either of the original TCRs it remains to be investigated whether pairing with other counterchains may result in a TCR with superior avidity and retained specificity similar to what has been described for other chain-centric TCRs (39, 40).

In conclusion the TCRs described herein may warrant further (pre)clinical evaluation particularly in the context of melanoma and glioblastoma. Further studies may focus on the 2C/165 TCR due to its higher avidity that translated also into increased reactivity against HLA-C*07:01^+^/CSPG4^+^ tumor cell lines. Given that it might well be that MHC-restricted anti-CSPG4 TCR are more sensitive in contrast to anti-CSPG4 CAR-T cells, on-target off-tumor toxicity must be critically assessed before any clinical evaluation of this TCRs is initiated. In this context, it is encouraging that CD4^+^ T cell-mediated anti-CSPG4 responses have been detected in both healthy individuals and melanoma patients (14, 15), and that both CD8^+^ T-cell clones described herein were retrieved from the peripheral blood of a melanoma patient. None of these naturally occurring anti-CSPG4 T-cell responses were recognizably associated with autoimmunity. However, these observations do not rule out immune-related toxicity when large quantities of CSGP4-reactive T cells are transferred in the context of an adoptive T-cell transfer. This risk could be mitigated by integrating a suicide switch into the TCR construct or by transfection of T cells with mRNA encoding the CSPG4-reactive TCR in order to avoid long-term expression of the transgenic TCRs. Collectively, our data support the immunotherapeutic potential of targeting CSPG4 using a TCR and complements previous studies on CAR T cells directed against this antigen (16–19).

## Data availability statement

The original contributions presented in the study are included in the article/**Supplementary Material**. Further inquiries can be directed to the corresponding author.

## Ethics statement

Patient-derived cell lines were established with informed consent and in accordance with the local legislation and institutional requirements. Human blood products were acquired from the blood bank of the University Medical Center of the Johannes-Gutenberg university Mainz in accordance with the local legislation and institutional requirements.

## Author contributions

CW and KK designed the study, performed experiments and curated data. KK wrote the original draft. MFatho and EH performed ELISpot experiments and MFatho curated these data. SL, VL and MFatho derived the T-cell clones 11C/73 and 2C/165 from Ma-Mel-86 blood lymphocytes. MFaust generated one of the TCR expression constructs. AP established and provided the Ma-Mel-86 model. MT provided essential resources and contributed to the conceptualization of the study. TW acquired external funding and supervised the study. CW, MFatho, TW, and MT edited the draft manuscript. All authors contributed to the article and approved the submitted version.
