# Supplementary figures and images for: Targeting the melanoma-associated antigen CSPG4 with HLA-C*07:01-restricted T-cell receptors
Source: Front Immunol. 2023 Oct 2;14:1245559. doi: 10.3389/fimmu.2023.1245559 (PMC10577170; doi:10.3389/fimmu.2023.1245559)

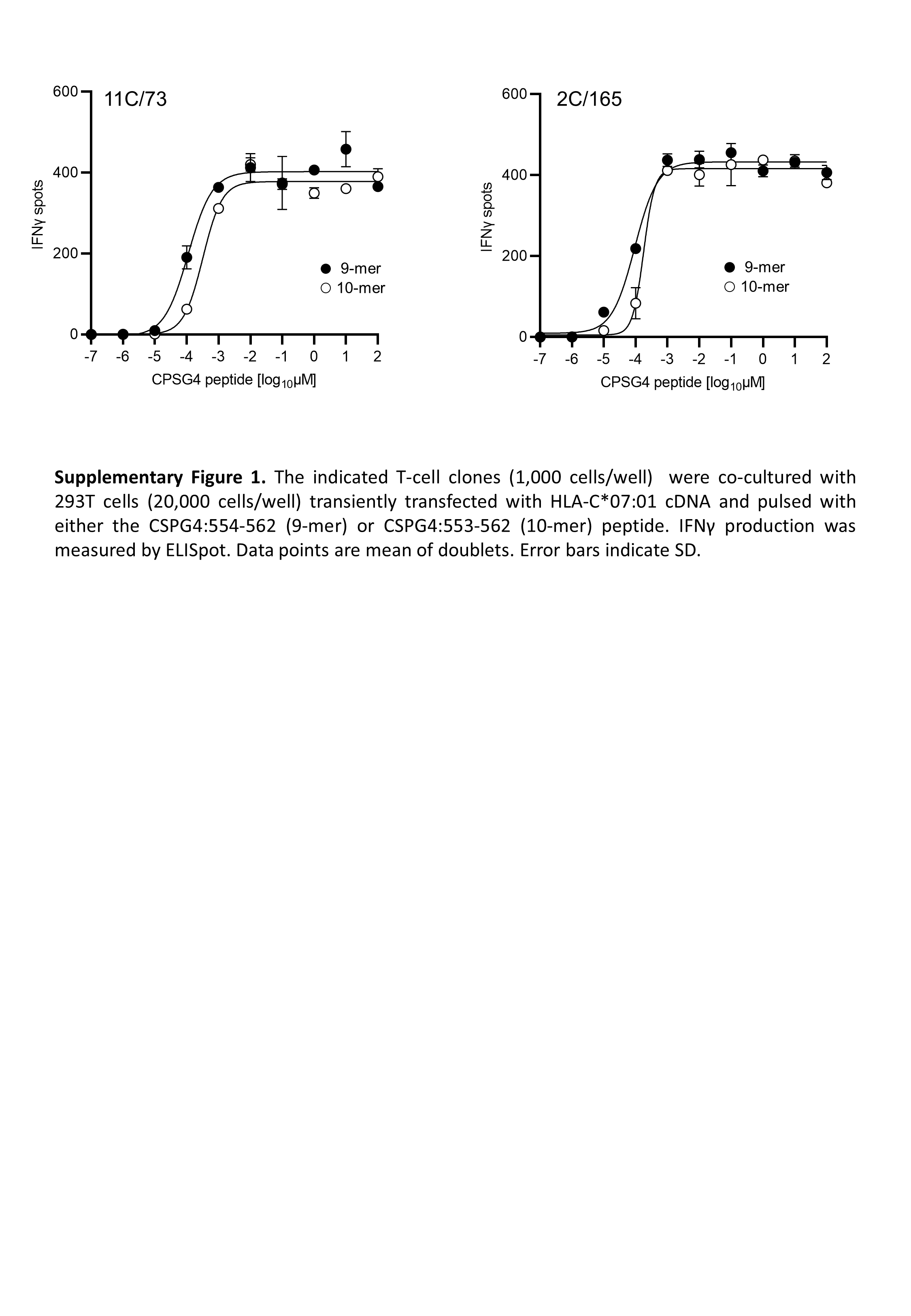

Supplement: Supplementary file 1 [file Image_1.tif]
